# Rapid two-dimensional characterisation of proteins in solution

**DOI:** 10.1038/s41378-019-0072-3

**Published:** 2019-07-01

**Authors:** Kadi L. Saar, Quentin Peter, Thomas Müller, Pavan K. Challa, Therese W. Herling, Tuomas P. J. Knowles

**Affiliations:** 1 Department of Chemistry, University of Cambridge, Lensfield Road, Cambridge, CB2 1EW UK; 2 Cavendish Laboratory, Department of Physics, University of Cambridge, J J Thomson Ave, Cambridge, CB3 0HE UK; 3 Fluidic Analytics Limited, Cambridge, UK

**Keywords:** Chemistry, Microfluidics

## Abstract

Microfluidic platforms provide an excellent basis for working with heterogeneous samples and separating biomolecular components at high throughput, with high recovery rates and by using only very small sample volumes. To date, several micron scale platforms with preparative capabilities have been demonstrated. Here we describe and demonstrate a microfluidic device that brings preparative and analytical operations together onto a single chip and thereby allows the acquisition of multidimensional information. We achieve this objective by using a free-flow electrophoretic separation approach that directs fractions of sample into an on-chip analysis unit, where the fractions are characterised through a microfluidic diffusional sizing process. This combined approach therefore allows simultaneously quantifying the sizes and the charges of components in heterogenous mixtures. We illustrate the power of the platform by describing the size distribution of a mixture comprising components which are close in size and cannot be identified as individual components using state-of-the-art solution sizing techniques on their own. Furthermore, we show that the platform can be used for two-dimensional fingerprinting of heterogeneous protein mixtures within tens of seconds, opening up a possibility to obtain multiparameter data on biomolecular systems on a minute timescale.

## Microfluidics: 2D fingerprints of heterogenous protein mixtures

Microfluidic devices provide a means for analysing heterogenous biomolecular mixtures rapidly, with high recovery rates and by using only very small sample volumes. Yet to date it has remained challenging to combine preparative and analytical processes onto a single chip and thereby fully realise the possibility of fast integrated analysis. Now, a team led by Tuomas Knowles from the University of Cambridge, demonstrates a chip that combines these two steps. Using voltage as the means to separate molecules, Kadi Saar and co-workers size a binary mixture of almost equally sized proteins which cannot be resolved by conventional sizing techniques. In parallel, the team uses the new device to acquire a 2D fingerprint of a heterogeneous protein mixture, opening up the possibility of obtaining multiparameter data on biomolecular systems on a rapid minute timescale.

## Introduction

Microfluidic platforms are attractive for the analysis of biological samples because of their very low sample consumption and high recovery rate^1–5^. Notably, microfluidic platforms can provide unsurpassed analysis speed both on the level of individual unit operations as well as on the level of a combined workflow as several functional units can be integrated directly without the requirement for transferring the sample between the units or for integrating connector elements or tubes—these transfer processes do not only extend the analysis process but also introduce dispersion, therefore affecting the performance of the system.

An important step in any workflow that involves working with heterogenous mixtures is the separation of components of interest, either to reduce the complexity of the mixture before it is directed to further processing or to purify it. Specifically, in the context of micron scale analysis, various continous flow based separation strategies have been explored and developed, such as free-flow electrophoresis, dielectrophoresis, magnetophoresis or acoustophoretic separation^6,7^. A common feature of these strategies is that the separation process occurs in the direction that is perpendicular to the direction of flow and it can, therefore, be performed continuously. This is in contrast to batch separation techniques, such as capillary electrophoresis where the analytes are separated in the same direction as the applied field. Although the latter approaches can be integrated with further processing^8–11^, their discontinuous operation limits the range and the complexity of the downstream steps that can be performed. Moreover, the use of a continuous separation strategy further allows performing the separation process under steady state conditions, which permits the intensity of the recorded signals and, thus, the sensitivity of the analytical downstream technique, to be increased.

A range of detection strategies have been integrated with microfluidic separation platforms with successful examples including laser induced fluorescence (LIF) or LED induced fluorescence^12–14^, chemiluminescence^15^, various electrochemical approaches^16–19^ and, despite the inherently short path lengths of micron scale channels, also UV absorbance^20^. Transferring these readings to concentrations, however, requires prior information about the components and, as such, the use of these techniques has remained limited to applications where the analytes in each specific fractions are already known or where their identities are subsequently determined with an offline technique. Integration of on-chip separation with analytical approaches beyond the detection of concentration, however, has been seldom achieved. For instance, the possibility to simultaneously determine the isoelectric points and concentrations of separated analyte molecules has been demonstrated^20^. However, this result was achieved through the inclusion of intrinsic calibration markers that enabled extracting quantitative information from the isoelectric focusing unit rather than through the integration of a downstream analytical unit, not serving as a general strategy for combined on-chip fractionation and analysis. Indeed, obtaining analytical information on the separated components beyond their concentration usually involves combining the separation with an offline analysis and identification strategy, most notably with mass spectrometry^21–23^ or with SDS-PAGE^24^. Such an offline analysis strategy, necessitating the requirement for sample transfer, eliminates one of the most attractive advantages of microfluidic technologies—its fast processing speed—and can further result in sample losses or contamination.

**Fig. 1:**
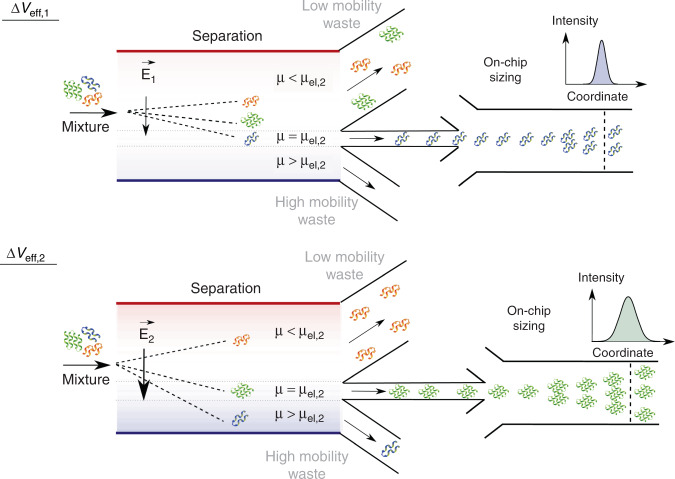
On-chip separation and analysis of mixtures in liquid phase. By adjusting the effective voltage, Δ*V*_eff_, molecules of a specific electrophoretic mobility, *μ* = *μ*_el_, and hence of a specific charge to hydrodynamic radius ratio, $\frac{q}{{R_h}}$, can be directed to the analysis area with those of smaller (*μ* < *μ*_el_) and larger (*μ* > *μ*_el_) mobility values directed to the waste collection channels. As the applied voltage can be varied, only a single analysis unit is required and the width of the separation chamber can be kept constrained, allowing the device to retain a high voltage efficiency and a fast processing speed. In the analysis area, the fractions are sized through microfluidic diffusional sizing (MDS) by monitoring their spatiotemporal motion under laminar flow conditions

To overcome these limitations and develop a strategy for fully integrated separation and quantitative characterisation of heterogenous biomolecular samples, here we developed a microfluidic device where an on-chip separation process was directly coupled to on-chip analysis—molecular sizing. Specifically, we used free-flow electrophoresis for fractionate analytes and, by varying the applied voltage, Δ*V*_eff_, directed molecules of a defined electrophoretic mobility, *μ*_el_, to the analysis unit (Fig. 1)^25^. This design feature permitted specific fraction to be directed to analysis by simply adjusting the applied field strength, which is in contrast to operational modes where a fixed gradient, such as pH (free-flow isoelectric focusing) or buffer mobility (free-flow isotachophoresis) is applied across the separation channel, which necessitates the use of a separate processing unit for each fraction. The use of an approach where the field strength could be varied, further allowed us to remove the requirement for separating the components out simultaneously. Instead, the fractions were directed to analysis sequentially, which enabled keeping the width of the separation channel constrained, thereby facilitating both narrower devices and, hence, faster processing speeds and the integration of stronger fields without increasing the applied potential. Downstream the separation area, we monitored the tempo-spatial movement of the molecules under flow which, due to the laminar nature of the flow, could be directly linked to their diffusion coefficients^26^. This allowed to a certain degree identifying the fractions that come out from the separation module, similarly to SEC-MALS (size exclusion chromatography with multi-angle light scattering) or LC (chip)-MS ((on-chip) liquid-chromatography–mass-spectrometry)^27^, with the additional benefits of performing the entire process in a fully integrated manner, on over an order of magnitude faster timescale and directly in-solution, where it becomes possible to probe dynamic biomolecular interactions that may disassemble in gaseous phase or in contact with a support medium. Moreover, in addition to enhancing the resolution of the sizing technique, the combined device enabled us to construct two-dimensional characteristic maps of the native charges and the hydrodynamic radii of the analytes—much like what could be obtained with two-dimensional electrophoresis gels but again, on orders of magnitude faster time scale and directly in the native environment of biomolecules. However, as the electrophoretic separation step was performed in free solution, the devices ensured that firstly, the map was obtained in the order of a few minutes which is orders of magnitude faster than the time-scale that could be reached with conventional and non-aqueous phase based techniques, opening up the possibility to study not only static but also dynamic systems. Secondly, the mixture was analysed in an environment where the molecules were not affected by the presence of the support medium, which unlike gel based methods permits the study of weak and non-covalent interactions that can be affected by the support medium.

## Results and discussion

### Device design

**Fig. 2:**
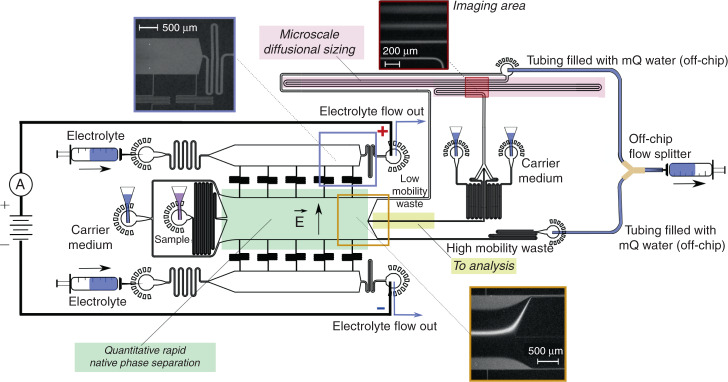
Device design and operation. A free-flow electrophoresis unit (green), allowing rapid separation of analyte molecules in their native phase and yielding quantitative information on the separation process, directed fractions of the sample (yellow) to a downstream analysis process involving microfluidic diffusional sizing (MDS; pink). The sample was characterised by monitoring a single imaging frame (top right inset) to simultaneously extract the sizes and the charges of the separated fractions. The device was operated by applying a negative pressure at its outlet with a Y-shaped off-chip flow splitter keeping the solutions from the “low mobility waste” and “high mobility waste” channels separated to avoid partial short circuiting of the device. The electric potential was applied from the electrolyte solution outlets employing a flowing electrolyte solution as described previously^13^. The flow of the electrolyte solution, which included a fluorescent tracer for its visualisation, was chosen such that the electrolyte would reach its outlet rather than be withdrawn into the separation chamber without reaching it (top left inset), yet it would leak into the chamber by a controlled distance, leaving sufficient space for deflecting the sample beam (bottom inset)

The device involved a native phase quantitative electrophoresis unit connected in series with a microfluidic diffusional sizing (MDS) unit (Fig. 2)^26^. This combined platform directed a component of a specific electrophoretic mobility, *μ*_el_, to on-chip downstream analysis as a function of the applied electric field strength. The device was designed such that with no field applied across the separation unit none of the sample molecules flowed into the analysis area. This objective was achieved by designing the three channels downstream of the electrophoresis unit (“low mobility waste”, “analysis”, and “high mobility waste”) to have different hydrodynamic resistances with ca. 64% of the flow directed towards the “low mobility waste” channel, around 5% to the analysis area and the rest to the “high mobility waste” channel. A variety of approaches has been demonstrated for integrating electric fields with micron scale channels to perform the free-flow zone electrophoresis separation process^7,28^. Here, we used our previously described strategy where the electric field was applied outside the microfluidic chip as it allowed strong electric fields to be applied in a stable manner by flowing any generated electrolysis products away from the chip without them entering the device^13^.

To facilitate device operation in a stable manner and as a result allow quantitative characterisation of the samples, we set out to minimise the number of individual units that drove the flow in the device. For this purpose, the outlets from individual channels were combined such that a single syringe could be used to apply a negative pressure at its outlet (Fig. 2)—this ensured that the flow in the individual channels was defined by the hydrodynamic resistances of the lithographically produced high-accuracy channels, in contrast to a system where the flows would be controlled by a number of external syringe pumps which could lead to relative fluctuations between the flows of the different fluids over time. The outlets of the electrolyte solution, however, were kept separated from the combined device outlet to (i) allow applying electric potential across the device without generating an electrical short-circuit and (ii) enable efficient removal of any generated electrolysis products without them accumulating and causing pressure fluctuations or cavitation at the joint outlet where the negative pressure was applied. Specifically, the electric potential was applied on metallic connectors outside the chip - this ensured that all gaseous products were generated on the metal and fluid interface outside the chip and not inside the microfluidic channels. The use a flowing liquid electrolyte solution (highly concentrated salt solution; “Materials and methods”), further ensured that the generated electrolysis products were flowed away from the chip while the applied field was propagated back to the chip instantaneously^13^.

The flow of the electrolyte to the electrophoresis chamber was controlled by narrow perpendicular channels (“bridges”) connecting the electrolyte channels to the electrophoresis area. These channels provided high hydrodynamic resistivity preventing the oppositely charged electrode solutions from coming into contact with each another while still allowing some of the electrolyte to flow to the electrophoresis area and through this provide direct fluidic and hence electrical connectivity between the electrophoresis chamber and the electrolyte channels. To maximise the efficiency of the electric field these channels were required to have a high hydraulic resistance R_HD_^29^ per electrical resistance R_EL_:

1 $$\frac{{{\mathrm{R}}_{{\mathrm{HD}}}}}{{{\mathrm{R}}_{{\mathrm{EL}}}}} = \frac{{\alpha (\gamma ) \cdot {\mathrm{R}}_{{\mathrm{HD}}}^ \ast }}{{\rho _{{\mathrm{el}}} \cdot \frac{{\mathrm{L}}}{{\mathrm{A}}}}}$$

where *ρ*_el_ is the resistivity of the electrolyte, $R_{\mathrm{HD}}^ \ast = \frac{{\eta L}}{{A^2}}$ is the hydraulic resistance of the channel with *η* being the viscosity of the fluid in it and A its cross-sectional area, *α*(*γ*) is the geometrical correction factor defined as $\alpha = \frac{{\pi ^3}}{8}\gamma ^2{\mathrm{f}}(\gamma )$ as a function of the aspect ratio of the channel $\gamma = \frac{{\mathrm{w}}}{{\mathrm{h}}}$ and w, h and L are the width, height and the length of the channel, respectively. After substitution and simplification we obtain:

2 $$\frac{{{\mathrm{R}}_{{\mathrm{HD}}}}}{{{\mathrm{R}}_{{\mathrm{EL}}}}} = \frac{{\pi ^3\eta }}{{8\rho _{{\mathrm{EL}}}{h}^2}}\gamma {\mathrm{f}}(\gamma )$$

With the height of the channel being fixed by the requirement for collecting a sufficient amount of signal for imaging (it was set to *h* = 50 μm), Eq. (2) is maximised when *γ*f(*γ*) is maximised. For a rectangular channel f(*γ*) is defined as^29^

3 $${\mathrm{f}}(\gamma ) = \left[ {\mathop {\sum}\limits_{{n} = 1,3,5...}^\infty {\frac{{{n}\gamma }}{{\pi {n}^5}} - \frac{2}{{\pi ^2{n}^5}}{\text{tanh}}({n}\gamma \pi )} } \right]^{ - 1}$$

from which we realise that *γ*f(*γ*) becomes maximal in the limit of *γ* → 0. Due to fabrication of lithographic channels becoming challenging when the widths of the produced lithographic structures are significantly smaller than their heights, we fixed the width of the connecting channels to *w* = 18 μm, at which it was still possible to produce lithographic structures with the photoresist that we used (“Materials and methods”).

We noted that during the operation of the device, the small amount of electrolyte solution that was designed to flow to the electrophoresis channel and generate a wall at the edges of the chamber (Fig. 2, bottom inset) ultimately reached the combined outlet, bringing the two oppositely charged electrolyte solutions into contact with one another and thereby reducing the potential across the electrophoresis chamber. In order to circumvent such partial short circuiting, we used a Y-shaped flow splitter to keep the streams apart until they had reached the splitter (Fig. 2). Crucially, the tubing connecting the two streams was filled with distilled water (18 MΩ cm^−1^) and its length was chosen such that the streams would stay non-connected during the operation of the device (Supplementary Materials Table S1 and Section S3).

### Fluid flow in the device

The device was designed such that at the end of the electrophoresis chamber, the fluids would split in a 13:1:6 ratio (64, 5, 31%), ensuring that no sample molecules flowed to the analysis area when no electric potential was applied across the separation chamber. Furthermore, the sample flow to the electrophoresis unit was designed to be in around 1:25 ratio with the carrier medium and in around 1:10 ratio in the diffusional sizing unit. The “bridges” between the electrophoresis area and the electrolyte channels were designed such that the device would retain as high voltage efficiency as possible while the flow of the electrolyte into the separation area would be minimised, leaving as much area as possible available for the separation process (Eq. (1)). The flow rates in each of the individual channels were then estimated by solving a set of simultaneous equations describing the mass balances and pressure drops in the device (Supplementary Materials Section S1, Eqs. (S.1)–(S.8). The corresponding flow rates in each of the channels are summarised in Supplementary Table S1.

We observed that the flow rate of the electrolyte into the device had a notable effect on the device performance. When the electrolyte infusion rate was low, the electrolyte solution did not reach the end of the channel (Supplementary Fig. S1b), which can generate a reversed flow from the electrolyte outlet back to the electrolyte channel, ultimately resulting in some of the electrolysis products entering the chip. Indeed, a negative flow in the electrolyte outlet channel was predicted when solving the system of Eqs. (S.1)–(S.8) to theoretically estimate the flow rates in the different channels at this infusion rate (Supplementary Materials Section S2). At sufficiently high flows, the electrolyte reached its outlet (Fig. 2, top right inset) and generated thin stable walls at the electrophoresis chamber walls (Fig. 2, bottom inset) as desired. This infusion rate was chosen for all further experiments. At high infusion rates, a significant amount of electrolyte was observed to leak to the main chamber, leaving only a very small area available for deflection and not permitting the sample molecules to be directed to the analysis area (Supplementary Fig. S1c). Indeed, when estimating the individual flow rates in the channels under these conditions, we found over 60% of the flow in the electrophoresis chamber to be comprised of the leaking electrolyte (Supplementary Materials Section S2).

### On-chip microfluidic diffusional sizing (MDS)

**Fig. 3:**
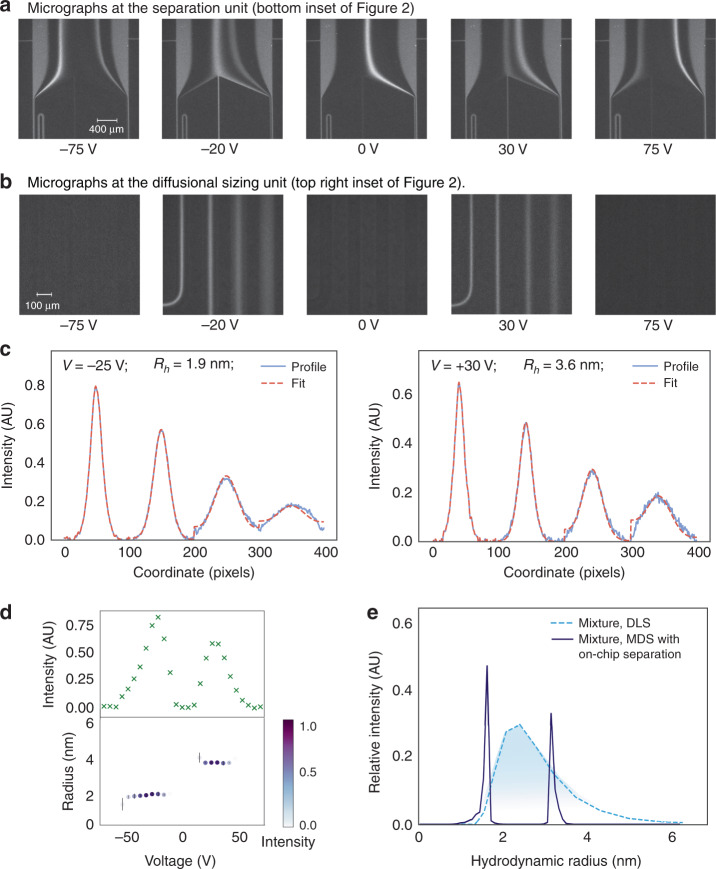
The sizing of a mixture that could not be deconvoluted using standard sizing techniques. **a** The voltage applied across the electrophoresis chamber was adjusted in linear steps to direct specific fractions to analysis. **b** The fractions were then sized by imaging the diffusional sizing unit consisting of four channels in which the extent of the diffusion of the analyte molecules into their surrounding carrier buffer was monitored. **c** The average size of the analytes molecules in each of the fractions were found by fitting the observed fluorescent profiles for different hydrodynamic radii (*R*_h_) and minimising the least mean square error between the fit (red dotted line) and the data (blue continuous line). **d** The fluorescence intensity in the analysis area varied depending on the concentration of the analytes in each of the fractions (top) and the sizes of the components in a binary mixture of lysozyme and bovine serum albumin were found to be *R*_h_ = 1.9 nm and *R*_h_ = 3.6 nm (bottom). **e** This binary mixture could not be characterised using sizing techniques that do not involve pre-fractionation steps, such as dynamic light scattering (DLS; light blue dotted line). Its sizing was possible using the device described in this work (dark blue continuous line)

The separation unit directed a fraction of the fluid flow to a MDS unit which similarly to analytical light scattering setups connected to size exclusion columns (SEC-MALS) allowed to a certain extent the identification of the fractions leaving the separation module. The diffusional sizing process relied on surrounding the analyte molecules with carrier medium and monitoring their diffusion into the medium in space and in time as described earlier^26^. As the sizing measurement had to performed for all applied voltages separately (Fig. 3a, b), we adjusted the strategy to ensure that only a single image would be sufficient for performing the sizing analysis (Fig. 2, red highligted area). Moreover, to analyse the data and obtain the average molecular size for each of the fractions, we replaced the Langevin dynamics based code used to model the movement of individual particles in the microfluidic channels^30^ with a numerical solver that enabled the predicted particle distributions to be generated significantly faster. These predicted distributions were then, as before, compared to the experimental data to extract the hydrodynamic radii of the particles in each of the fractions.

The positions of the locations used for imaging (Fig. 3b) were chosen such that the first one would be as close as possible to the nozzle where the sample meets the carrier medium - this placement allowed its usage as a reference point from which the movement of the particles was propagated. The position of the furthest away point was chosen such that for a representative protein with *R*_h_ = 3 nm (*D* = 7 × 10^−11^ m^2^ s^−1^), the molecules would have diffused away from the centre of the channel but would have not yet become uniformly distributed across it—such placement enabled us to maximise the amount of information that could be extracted from the profiles. To enable accurate sizing of analyte molecules that are orders of magnitudes larger or smaller in size, the length of the diffusional sizing channel or the flow rate can be adjusted accordingly. Last but not least, in order to eliminate any effect from the autofluorescence of the PDMS-based devices, originating principally from the additives included in the cross-linking agent, a background image with no sample flowing in the device was also recorded and subtracted from the data before the images were analysed.

### Analysis of a binary protein mixture

We used the device to analyse a binary mixture of two sample proteins—bovine serum albumin (BSA; *M*_w_ = 66 kDa) and human lysozyme (*M*_w_ = 15 kDa). To preserve the protein molecules in their native states and enable a label-free analysis of the sample, the imaging was performed with a home-built UV-wavelength based microscope that relied on recording the intrinsic fluorescence of the sample by exciting their tryptophan and tyrosine residues (“Materials and methods”). We first confirmed the ability to separate the mixture into its components by applying a set of voltages and recording the fluorescent profiles (Fig. 3a). We then operated the device by applying a voltage ramp from −75 to 75 V and instead of the separation area, recorded the profiles downstream at the diffusional sizing area containing four sections of the channel as described in Section 3.3. With electrophoretic mobilities, *μ*_el_, of proteins being in the order of O ~ 10^−8^ m^2^ V^−1^ s^−1^, this voltage range, in combination with the used flow rate of around 200 μL h^−1^, allows for the characterisation of most proteins and their complexes. Alternations to the flow rate or to the applied voltage range can be introduced if biomolecules with different biophysical parameters are to be analysed. Moreover, the applied voltage step gives a possibility to adjust the resolution. It can even be applied in a non-linear manner to provide increased accuracy in specific electrophoretic mobility ranges.

To precisely locate the imaging position the images were recorded at a position where the corner of the first channel would be visible (Fig. 2 red square; Fig. 3b) with only the part of the frame where the channels appeared straight used for the sizing analysis. The best fits to the individual profiles were used to extract the hydrodynamic radii at each of the voltages (Fig. 3c) and were found to be around 1.9 nm (at −20 V) and 3.6 nm (at 30 V) which are in agreement with the values obtained by using dynamic lights scattering (DLS; Supplementary Fig. S2). The extracted size was robust and was not affected by the intensity and the concentration of the molecules in the analysis area (Fig. 3d).

The characterisation of mixtures which include molecules of similar properties is known to be challenging because it requires deconvolution of an average signal, which is a difficult inverse problem^26,31^. As such, polydisperse mixtures are commonly analysed by first physically separating the individual components within a mixture, for instance, by gel filtration or a type of chromatography, such as liquid chromatography or electrochromatography, and only then detecting and sizing the individual fractionated species, for example, by absorbance or by light scattering. Indeed, when examining the mixture of BSA and human lysozyme using DLS, the presence of averagely sized molecules rather than that of the individual species was observed (Fig. 3e, light blue dashed line).

Mixtures of nanoscale molecules where the individual analytes are of similar size but exhibit differences in their electrophoretic properties, can be rapidly characterised using the device described here. Indeed, representing the obtained hydrodynamic radii (Fig. 3d) on a histogram allows the size distribution of this sample to be characterised and it confirms the presence of two distinct components (Fig. 3e, dark blue continuous line). Whereas a similar result could have been obtained using an off-chip separation approach, the latter strategy would have introduced the requirement for fractionation and for transferring the sample from one analytical tool to another or integrating connector elements or tubes, and could have thus been performed only over a significantly longer timescale.

The measurements were performed using protein concentration of the order of 100 μM. The data in Fig. 3d incidate that the sizing step can be performed effectively at close to an around an order of magnitude lower concentrations with the current setup. In general, we have shown that relying on their intrinsic fluoresence, proteins can be sized accurately down to a sensitivity limit of around 100 nM^32^. Crucially, the analysis described in this manuscript can be performed using alternative optical detection approaches, including through the use of fluorescently labelled samples to increase the sensitivity or even with single-molecule detection approaches if the ultimate sensitivity limit is required^33^. Moreover, in the case of optically non-active compounds, alternative detection and characterisation approaches could be used, such as dry mass sensing^34^.

### Two-dimensional fingerprinting of protein mixtures

Finally, we showed that the described strategy can be used to obtain two-dimensional characteristic maps on this protein mixture. To extract quantitative information from the separation step and specifically, to relate the applied potentials to the electrophoretic mobilities of the species that were directed to the analysis area at this voltage, we estimated the voltage efficiency of the device. Specifically, by recording the currents flowing in the system both, during the normal operation mode and when the separation chamber was short-circuited, we obtained estimates for the total electrical resistance of the device and that of the electrodes to be *R*_device_ = 644 kΩ and *R*_electrodes_ = 521 kΩ (Supplementary Materials Section S4), indicating that at each of the applied potentials around 14% of it drops across the separation chamber.

The movement of a particle in electric field can be related to its electrophoretic mobility though $\mu _{\mathrm{el}} = \frac{{v}_{\text{drift}}}{E}$, where *v*_drift_ is the drift velocity of the analyte in the electrophoresis chamber and E the strength of the electric field across the chamber. We noted that the electric field applied here across the channel varied slightly as a function of the distance along the electrophoresis chamber due to the additional amount of electrolyte that flowed in from the side channels, slightly decreasing the effective separation between the two electrodes. Under these circumstances the electrophoretic mobility can be expressed as

4 $$\mu _{{\text{el}}} = \frac{{{v}_{{\text{drift}}}}}{{E}} = \frac{{\frac{\delta }{{{t}_{{\text{res}}}}}}}{{\frac{{{V}_{{\text{eff}}}}}{{w}}}} = \frac{{\delta \cdot {Q} \cdot {w}}}{{({w} \cdot {h} \cdot {L}) \cdot {V}_{{\text{eff}}}}} = \frac{{\delta \cdot {Q}}}{{{h} \cdot {L} \cdot {V}_{{\text{eff}}}}}$$

where *V*_eff_ is the effective voltage across the separation chamber, *δ* is the observed deflection and *Q* is the flow rate in the separation area (the sum of the carrier medium flow *Q*_cm_ and the sample flow *Q*_s_). As described, the device used in this work was designed such that the molecules observed in the analysis area were those deflected away from their original position by around 15% of the total width of the separation chamber or by around 300 μm.

**Fig. 4:**
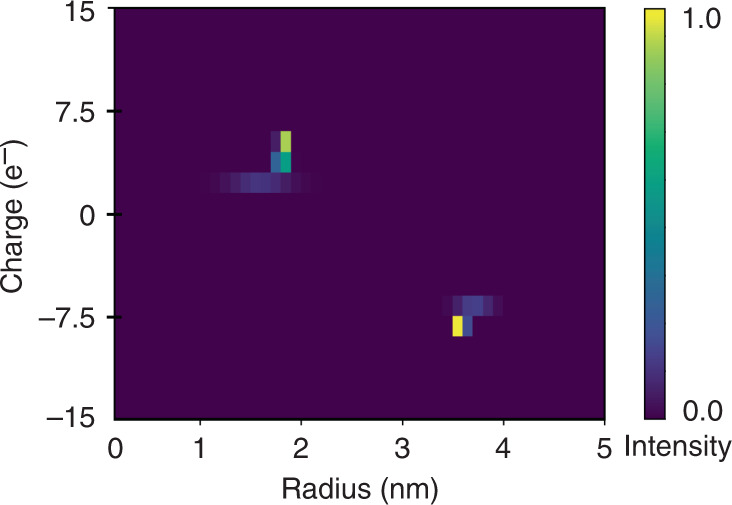
Rapid two-dimensional characterisation of a mixture of bovine serum albumin and human lysozyme. The quantitative nature of the eletrophoretic separation unit allowed the applied potential to be related to the electrophoretic mobility *μ*_el_ of each of the fractions. These data were used in combination with the extracted hydrodynamic radii (*R*_h_) to estimate the effective charges (*q*) of the molecules in each of the fractions and to construct a two-dimensional *q* vs *R*_h_ map of the mixture over a few minute timescale by monitoring the device only at a single imaging frame

Using Eq. (4), we estimated the electrophoretic mobilities of each of the fractions. Crucially, electrophoretic mobility is known to depend on the charge to hydrodynamic radius ratio of the analyte, $\mu _{{\mathrm{el}}} = \frac{{q}}{{{kT}}}{D}$. Hence, having obtained an independent estimate for the diffusion coefficients, we can now use the obtained electrophoretic mobility values to estimate the native charges of the molecules in each of the fractions. Based on these data, we constructed a two-dimensional characteristic map of the effective charge (*q*) and hydrodynamic radius (*R*_h_) of the mixture (Fig. 4). The native charges of the BSA and the lysozyme molecules can be seen to be around −9 and +6 elementary charge units, respectively, which are in agreement with the values estimated using other techniques^35–38^.

Crucially, the full two-dimensional map was obtained from monitoring only a single imaging frame, setting the basis for rapid in solution analysis. Indeed, the time period for the analyte molecules to move from through the separation and the diffusional sizing areas and to be further imaged was around 14 s. Thus, the fractions could be analysed at a rate of 4 fractions per minute. In particular, in our current experiments, the two-dimensional map (Fig. 4) was constructed using only 3 μL of sample and over a 7 min time-period, which is orders of magnitude faster than the timescales over which conventional two-dimensional protein gels are performed. The resolution limit of the system depends on the resolution of the upstream separation process and was estimated to be around three elementary charge units for our system (Supplementary Materials Section S5). This performance allows a wide of biomolecular interactions to be probed directly in solution, under their native conditions, including the binding of small molecules and certain metal ions to proteins and their complexes, which are studies challenging to perform outside the native environment of proteins.

## Conclusions

Current microscale on-chip separation approaches commonly rely on collecting the separated fractions and sending these to offline processing rather than analysing them directly on chip. This undermines one of the key advantages of microscale platforms—their fast analysis speed—and may also introduce the requirement for additional fluid handling steps. We have demonstrated a microfluidic device that combines on-chip separation with direct on-chip analysis—spatiotemporal diffusional sizing. We used the device to analyse a binary mixture of proteins that cannot be identified as individual components by existing solution sizing approaches. Moreover, the quantitative nature of the separation process further allowed us to construct a two-dimensional characteristic map of this heterogeneous mixture on a few minute timescale, opening up the possibility of rapidly characterising mixtures directly in solution and at time resolutions not accessible with current biophysical techniques.

## Materials and methods

### Fabrication of microfluidic devices

The device was designed using AutoCAD software (Autodesk) and printed on acetate transparencies (Micro Lithography Services). The replica mould for fabricating the device was prepared through a single, standard photo-lithography step^39^ by spinning SU-8 3050 photoresist (MicroChem Corp.) onto a polished silicon wafer to a height of 50 μm. The UV exposure step was performed with a custom-built LED-based apparatus^40^ and the precise heights of the features measured by a profilometer (Dektak, Bruker). The mould was then used to generate poly(dimethylsiloxane) (PDMS; Dow Corning) based chips. The channels on the chips were sealed with a quartz slide (Advalue Technology, 76.2 × 25.4 × 1.0 mm) after their surfaces had been activated through an oxygen plasma (Diener electronic, 40% power for 15 s). Before injecting the solutions into the channels, the chips were exposed to an additional plasma oxidation step (80% power for 500 s) which rendered the channel surfaces more hydrophilic^41^.

### Sample preparation

BSA and human lysozyme were purchased from Sigma Aldrich and used without further purification. The experiments were performed with the proteins, 2 mg mL^−1^ BSA and 5 mg mL^−1^ lysozyme, dissolved in 10 mM sodium phosphate buffer at pH 7.4. The buffer and the protein solutions were filtered before experiments (EMD Millipore^TM^, Millex^TM^, 0.22 μm).

### Device operation and calibration

The device was primed from its outlet with distilled water, following which gel loading tips (Fisherbrand^TM^, 1–200 μL) filled with the sample and the relevant carrier media were inserted into their respective inlets. The device was operated by applying a negative pressure at its outlet using a 1 mL glass syringe (Hamilton®) connected to a syringe pump (Cetoni neMESYS) set to operate at 500 μL h^−1^. The syringe was connected to an off-chip Y-shaped connector (IDEX Health & Science) to keep the solutions from the two outlets of the device separated. The electrolyte solution (3 M KCl with 1 mg mL^−1^ BSA) was injected into its corresponding inlets similarly using 1 mL glass syringes. The electric potential was applied using a 500 V bench power supply (Elektro-Automatik EA-PS 9500-06) that had its terminals connected to hollow metal dispensing tips (20G, Intertonics) and inserted to the outlets of the electrolyte channels. The potential was varied in linear steps, images recorded using a deep-UV fluorescent microscope and current readings concurrently using a digital multimeter (Agilent Technologies 34401A, Santa Clara, CA). The measurements for determining the electrical resistance of the electrodes and estimating the effective electrical potential applied across the devices were performed in an identical manner but with the gel loading tips at the sample and carrier medium inlets replaced with those filled with 3 M KCl solution as has been described in detail earlier^13^. All measurements were performed at room temperature.

### Optical detection in the deep UV-wavelength region

The movement of the protein molecules in the microfluidic chips was visualised using an inverted deep-UV fluorescence microscope as described earlier^32^. Briefly, the sample was illuminated using a 30 mW 280 nm LED (Thorlabs) exploiting the intrinsic fluorescence of aromatic residues of proteins in the deep-UV wavelength range. The light was passed through an aspherical lens of a focal length of 20 mm to get a nearly collimated beam and after this onto a dichroic filter cube (280/20–25 nm excitation, 357/44–25 nm emission, 310 nm dichroic beamsplitter). The reflected light from the dichroic mirror was focused onto the sample by an infinity corrected UV objective lens (Thorlabs LMU-10X-UVB; numerical aperture of NA = 0.25) and the emitted light collected through the same objective, passed through the emission filter and focused onto an EMCCD camera (Rolera EM-C2). All the used optics were made out of fused silica to enable high transmission in the UV wavelength region.

## Supplementary information

Supplementary Materials
