# Supplementary material for: Rapid two-dimensional characterisation of proteins in solution
Source: Microsyst Nanoeng. 2019 Jul 1;5:33. doi: 10.1038/s41378-019-0072-3 (PMC6799820; doi:10.1038/s41378-019-0072-3)
Supplement: Supplementary file 1 — Supplementary Materials [file 41378_2019_72_MOESM1_ESM.pdf]

Rapid two-dimensional characterisation of proteins in-solution - Supplementary Information

Kadi L. Saar,[1] Quentin Peter,[1,2] Thomas Müller,[3] Pavan K. Challa,[1,2] Therese W. Herling,[1] and Tuomas P. J. Knowles*[1,2]

[1]*Department of Chemistry, University of Cambridge, Lensfield Road, Cambridge CB2 1EW, UK*

[2]*Cavendish Laboratory, Department of Physics, University of Cambridge, J J Thomson Ave, Cambridge CB3 0HE, UK*

[3]*Fluidic Analytics Limited, Cambridge, UK*

S1 Estimating the flow rates in the device

The flow rates in the individual channels of the device were estimated by solving the following set of simultaneous equations:

$$Q_{cm} + Q_s + 2 \cdot Q_d + 2 \cdot Q_{el,in} = Q_{out} + 2 \cdot Q_{el,out} \tag{S.1}$$

$$2 \cdot Q_d + Q_{lm} + Q_a + Q_{hm} = Q_{out} \tag{S.2}$$

$$2 \cdot Q_d + Q_a = Q_{sizing} \tag{S.3}$$

$$R_{cm} \cdot Q_{cm} = R_s \cdot Q_s \tag{S.4}$$

$$R_{bridge} \cdot \frac{(Q_{el,in} - Q_{el,out})}{N} = R_{el,out} \cdot Q_{el,out} + R_{cm} \cdot Q_{cm} \tag{S.5}$$

$$R_{lm} \cdot Q_{lm} = R_{hm} \cdot Q_{h,} \tag{S.6}$$

$$R_{lm} \cdot Q_{lm} = R_a \cdot Q_a + R_{sizing} \cdot Q_{sizing} \tag{S.7}$$

$$R_{cm} \cdot Q_{cm} + R_a \cdot Q_a = \frac{1}{2} R_d \cdot Q_d \tag{S.8}$$

where Q_i and R_i correspond to the flow rate in channel i and to the hydraulic resistance of that channel, respectively, as indicated in Supplementary Figure 1. Equations (S.1)-(S.3) describe the mass balances for the total device, for the analysis area downstream of the separation unit and for the diffusional sizing unit, respectively; equations (S.4)-(S.5) equate the pressure drop between points A and B; equations (S.6)-(S.7) the pressure drop between points B and D and equation (S.8) the pressure drop between points A and C.

The flow rates in each of the channels under the conditions used to operate the device ($Q_{out} = 500\ \mu L\ h^{-1}$ and $Q_{el,in} = 80\ \mu L\ h^{-1}$) estimated by solving the set of simultaneous equations (S.1 - S.8) are summarised in Supplementary Table 1.

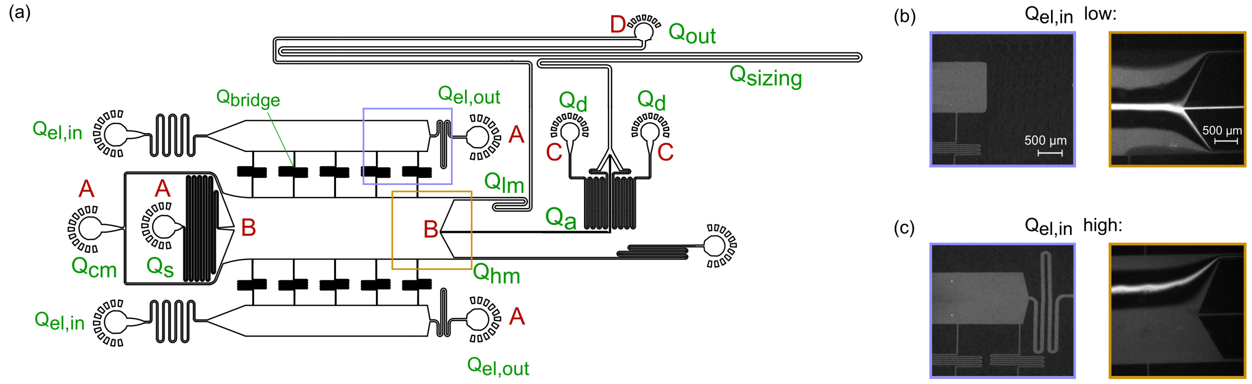

Figure S1. **(a)** The flow rates in each of the channels were estimated by solving the system of simultaneous equations (S.1 - S.8). **(b)** At low relative electrolyte flows ($Q_{out} = 500\ \mu L\ h^{-1}$, $Q_{el,in} = 30\ \mu L\ h^{-1}$) the electrolyte solution was observed not to reach the electrolyte outlet (left) but to get withdrawn into the separation chamber, not enabling the propagation of the electric potential from the electrolyte outlet back to the device (right). **(c)** At high relative electrolyte flows ($Q_{out} = 500\ \mu L\ h^{-1}$, $Q_{el,in} = 200\ \mu L\ h^{-1}$) the electrolyte reached its outlet (left) but leaked too far into the separation chamber to permit directing the sample molecules into the analysis area (right).

Table S1. The flow rates of the solutions in the channels of the device with the withdrawal flow rate from the device outlet being $Q_{out} = 500\ \mu L\ h^{-1}$ and the electrolyte infusion rate into the device being $Q_{el,in} = 80\ \mu L\ h^{-1}$.

**Channel**	**Symbol**	**Width** (μm)	**Length** (mm)	**Pressure drop** (Pa)	**Flow rate** ($\mu L\ h^{-1}$)
Carrier medium to the electrophoresis unit	Q_{cm}	50	5.1*	603	188*
Sample	Q_s	40	40.5	603	7.4
Carrier medium to the sizing unit	Q_d	50	8.6*	730	135
Low mobility waste	Q_{lm}	60	10.2	1561	172*
High mobility waste	Q_{hm}	50	15.5	1561	80
To analysis	Q_a	30	2.5	128	13
Diffusional sizing channel	Q_{sizing}	100	25.0	1432	148
Electrolyte out	$Q_{el,out}$	60	4.5	101	25
Bridge	Q_{bridge}	18	6.9	704	7

* Two parallel channels of that length. The flow rate is given as the combined flow rate from the two channels.

S2 The effect of the electrolyte infusion rate on the device performance

We observed that when the electrolyte infusion rate into the system is low, the solution gets withdrawn into the main chamber without reaching its outlet (Supplementary Figure 1b). Specifically, at electrolyte infusion rate of $Q_{\mathrm{el,in}} = 30\ \mu\mathrm{L\ h^{-1}}$, by solving the system of equations (S.1-S.8), we estimate the flow at the electrolyte outlet channel to be $Q_{\mathrm{el,out}} = -10\ \mu\mathrm{L\ h^{-1}}$. However, when the electrolyte flow into the system is high, the electrolyte solution leaks far into the separation channel. Specifically, at the infusion rate of $Q_{\mathrm{el,in}} = 200\ \mu\mathrm{L\ h^{-1}}$, again by solving the system of equations, we estimate that the electrolyte leaks in from all the bridges at a rate of $Q_{\mathrm{bridge}} = 25\ \mu\mathrm{L\ h^{-1}}$ or at a total rate of around $250\ \mu\mathrm{L\ h^{-1}}$. Under these conditions the combined flow of the carrier medium and the sample into the electrophoresis chamber is $Q_{\mathrm{cm}} + Q_{\mathrm{s}} = (201 + 8)\ \mu\mathrm{L\ h^{-1}} = 209\ \mu\mathrm{L\ h^{-1}}$, indicating that over half of the separation chamber is filled with the electrolyte (Supplementary Figure 1c).

S3 Flow splitting at the device outlet

In order to avoid partial short-circuiting of the device at its outlet, we used a Y-shaped flow splitter that would prevent the oppositely charged electrolyte streams coming into contact with each other at the device outlet but only further downstream (Figure 2 in the Main Text). The tubing on each side of the Y-shaped splitter was $\mathrm{L} = 40\ \mathrm{cm}$ long and $\mathrm{D} = 0.86\ \mathrm{mm}$ in its internal diameter, and the flow rates of the solutions into its two sides around 320 μL h^{-1} and 80 μL h^{-1} (Supplementary Table 1). It would therefore take around

$$t = \frac{\pi \cdot (\frac{D}{2})^2}{Q} \tag{S.9}$$

$t_1 = 43$ min and $t_2 = 170$ min for the fluid to reach the flow splitter from the two sides. Even when accounting for Taylor dispersion in the tubing, this time scale is significantly longer than the imaging period ($\sim$ 7 minutes) and as such the voltage efficiency of the device can be assumed to remain unaffected throughout its operation.

S4 Estimating the effective voltage drops across the separation chamber

To estimate the effective voltage drop across the separation chamber, simultaneously with applying the voltages, we also recorded the currents flowing in the system. This allowed us to estimate the resistance of the micron scale device to be $R_{device} = 644$ kΩ. To obtain an estimate for the electrical resistance of the electrodes, we filled the device with a highly conductive solution (3M KCl) to short-circuit it. By doing so, we obtained an estimate of $R_{electrodes} = 521$ kΩ for the resistance of the electrodes. These data indicate that at each of the applied voltage, around 14% of it drops across the separation chamber.

S5 Resolution of the device

The resolution of the setup depends on the extent of beam broadening that the analyte molecules undergo when moving down the separation area. With the collection area for analysis being around 15% of the total width of the separation chamber away from its centre, at this deflection the analyte beams have a width of about 180 μm (Figure 3a, Main Text; defined as two standard deviations). Thus, our demonstrated setup can resolve two analytes when the difference in their deflected distances is least 180 μm + 0.05 · 2000 μm

$= 280\ \mu$m, where the coefficient 0.05 describes the fraction of fluids that enters the analysis area. At the maximum electric field applied in our experiments, this corresponds to an electrophoretic mobility difference of about $1 \cdot 10^{-8}$ m^2 V^{-1} s^{-1}. For a representative protein molecule with $R_h = 3$ nm, this corresponds to a charge change of about three elementary units.

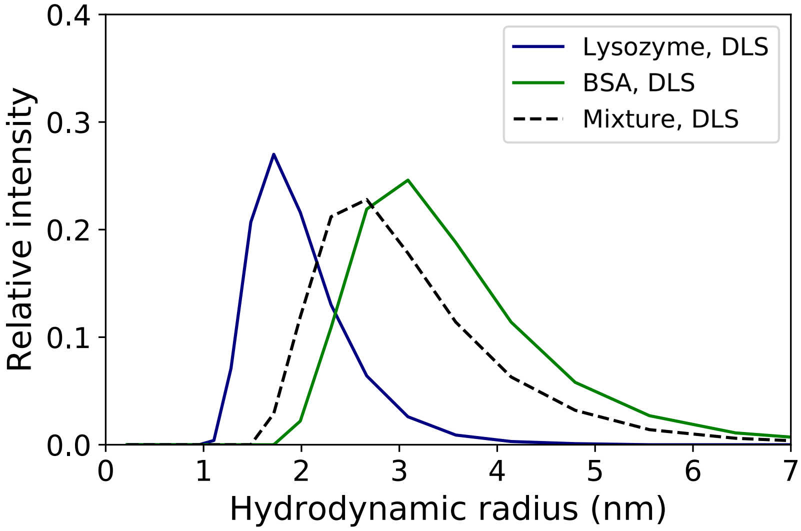

Figure S2. Sizing lysozyme (blue solid line), BSA (green solid line) and their mixture (black dotted line) using dynamic light scattering.
